# The Effect of Conversion from a Calcineurin Inhibitor to Sirolimus on Skin Cancer Reduction in Post-renal Transplantation Patients

**DOI:** 10.7759/cureus.1564

**Published:** 2017-08-13

**Authors:** Aaron Smith, Wei Niu, Anand Desai

**Affiliations:** 1 College of Medicine, University of Central Florida

**Keywords:** non melanoma skin cancer, kidney transplant, sirolimus, calcineurin inhibitor, kaposi sarcoma, renal transplant

## Abstract

In kidney transplant patients, skin cancer is the most commonly involved neoplasm. More than 90% of post-transplantation skin cancers are nonmelanoma skin cancers (NMSCs). The majority of them are squamous cell carcinomas and basal cell carcinomas. Calcineurin inhibitors (CNIs) such as cyclosporine and tacrolimus are immunosuppressive agents given after solid organ transplantation, but they can also promote tumor growth. Sirolimus is a novel class of immunosuppressants and has been proven to have antineoplastic properties. We review clinical trials and meta-analyses studying if conversion from CNI to sirolimus in post-renal transplantation patients decreases the development of NMSCs. A critical appraisal of the literature demonstrated that, while smaller scale studies tended to yield no clinically significant data, larger clinical trials and meta-analyses supported the conclusion that converting to sirolimus in post-renal transplant patients leads to reductions in skin cancer development. As a result, we conclude that conversion to sirolimus likely reduces NMSC in post-renal transplantation patients. Larger scale clinical trials with more rigorous stratification and less patient dropout rate are needed for more definitive conclusions.

## Introduction and background

Skin cancer occurrence is quite common after organ transplantation [1]. In kidney transplant patients, skin cancer is the most common secondary neoplasm, and 60-80% of them develop multiple subsequent skin cancers after the first cutaneous squamous cell carcinoma (SCC) [2]. More than 90% of post-transplantation skin cancers are nonmelanoma skin cancers (NMSCs). Among them, SCC and basal cell carcinoma (BCC) are the two major types of skin cancer. Other types of skin cancer include Bowen’s disease, Kaposi’s sarcoma, mixed squamous and BCC, and melanoma [3]. Compared with the age-matched general population, SCC in post-transplantation patients is 65 to 250 times more likely to occur, and its course is more aggressive [4-5]. In comparison, BCC is 10-20 times more likely to occur in post-transplantation patients [6-7]. The prevalence of skin cancer is directly correlated with the duration of immunosuppressive therapy [7-8].

Calcineurin inhibitors (CNIs) such as cyclosporine and tacrolimus have classically been used as standard immunosuppressive agents after solid organ transplantation. They work by selectively inhibiting calcineurin, which primarily suppresses the transcription of interleukin 2 (IL-2), T cell activation, and T cell-dependent B cell activation [9]. They particularly can improve short-term survival in renal allograft patients. However, their uses have not been associated with increased long-term survival, partially because of CNI nephrotoxicity [10-11]. CNIs can also promote tumor growth [12-13]. Because of the risk associated with CNIs use, there have been efforts to either identify other immunosuppressants or at least find strategies to minimize the toxicities of CNIs. Sirolimus is a novel class of immunosuppressants that inhibit the mammalian target of rapamycin, which is a serine-threonine kinase. This enzyme has an important regulatory function in cell growth and proliferation [14]. Via the effect on this enzyme, sirolimus exhibits immunosuppressive effects via inhibition of B cell and T cell proliferation. While it is tempting to presume that sirolimus can promote tumor development and progression via immunosuppression, sirolimus has been proven to have antineoplastic properties [15]. Interestingly, sirolimus inhibits cancer cell proliferation through the same mechanism that is responsible for immunosuppression [16]. Multiple clinical trials have shown that withdrawal of cyclosporine from a combination therapy of cyclosporine, sirolimus, and corticosteroids two to four months post-renal transplantation leads to improved renal function, less malignancy, and overall improved long-term survival and life quality [17-19].

Currently, most renal allograft patients receive CNIs as the initial immunosuppressive therapy. Some clinical trials examining post-renal-transplantation maintenance therapy involving conversion from CNI to sirolimus show improved renal function and survival within a two-year study period [20]. However, there is also conflicting evidence suggesting that, while conversion from CNIs to sirolimus is associated with improvement in glomerular filtration rate and reduction in risk of malignancy and NMSC, conversion is also associated with higher risk of rejection and mortality [21-22]. More long-term follow-up data on graft rejection and survival in renal transplant patients is required for evaluation. As for the risk of skin cancer, CNI withdrawal and conversion to sirolimus after renal transplantation have been associated with reduced risk of different types of NMSCs such as SCC, BCC, and Kaposi’s sarcoma in multiple clinical trials [23-25].

This review will summarize clinical trials that convert CNIs to sirolimus in renal transplantation patients as well as meta-analyses on the subject. The articles will be reviewed in order based on their type, starting with clinical trials and ending with three meta-analyses. The aim is to carefully weigh the delicate balance of the benefits and risks of conversion to sirolimus and explore the possibility of a larger scale clinical use of sirolimus to reduce skin cancer in renal transplant patients.

## Review

Clinical trials

Not many studies on post-transplant sirolimus therapy have examined the risk of Kaposi’s sarcoma in transplant recipients. A case series by Stallone, et al. looked at the effect of conversion to sirolimus in renal transplant recipients with biopsy proven Kaposi’s sarcoma [24]. The study included cadaveric kidney transplant recipients with biopsy proven Kaposi’s sarcoma who tested negative for HIV. There were 15 patients included in the trial. The average age was 48.7 ± 7.9 years, and 12 of the 15 patients were male. The patients were initially on cyclosporine and mycophenolate mofetil before they were diagnosed with Kaposi’s sarcoma. Each patient underwent an excisional biopsy of one lesion. After the diagnosis, these drugs were discontinued and the patients were started on sirolimus for immunosuppression. This trial did not include a control group. All patients were changed to sirolimus therapy.

After one month of sirolimus therapy, all of the patients underwent a skin examination. In 80% of the patients, the Kaposi’s sarcoma lesions visually started disappearing. After three months of therapy, all 15 patients showed no visual signs of any lesions. At six months each of the patients had a second excisional biopsy at the site of the previous biopsy to check for clinical remission. Each of the 15 patients had a negative second biopsy. While this study did not prospectively compare sirolimus to a control group, and had a small and non-diverse sample size, it provides preliminary evidence that sirolimus has an anti-tumor effect in kidney transplant recipients with proven Kaposi’s sarcoma.

Salgo, et al. performed a randomized, assessor blind, controlled study that examined if conversion to sirolimus immunosuppression in renal transplant patients decreased the rate of premalignant skin lesions and NMSC [25]. The study looked at 44 patients initially but only 33 were included in the analysis. There were 25 patients in the sirolimus group, but two participants withdrew consent and seven participants had adverse events. There were 19 patients in the control group, one of which was lost to follow up and one of which had adverse events. The attrition rate was thus 36% in the sirolimus group and 11% in the control group. The method for randomization was not stated, but baseline characteristics and clinical characteristics were similar between groups. Groups were largely treated equally. The authors report only one protocol deviation in which the seven-point scale used to assess premalignancies was not used in one patient’s one-year follow-up.

The study’s primary endpoint involved examining premalignant skin dysplasia at baseline then one year later. The assessment was completed by blinded dermatologists using a seven-point Likert scale that looked at multiple aspects of patients lesions, including number, size, etc. The secondary analysis examined the presence of new NMSCs that developed during the study period.

Overall, sirolimus proved to have better outcomes regarding skin lesions. Eleven (68.8%) patients had no change in skin lesion status and five (31.2%) patients had an improvement in skin lesions. In the control group, 12 (70.6%) patients had no change in skin lesion status and five (29.4%) patients had a worsening in their skin lesions. Analysis showed a statistically significant difference in progression of skin lesions between groups at six months (p < .0005) and 12 months (p < .0001). Nine individuals developed NMSC during the course of the study. Eight of these participants were in the control group and the one remaining participant was in the sirolimus group (p = .0167). The p-values of these findings provide evidence that sirolimus therapy may lead to a halt in the progression of NMSC when compared to other traditional therapies.

While the results of this study are promising that conversion to sirolimus improves NMSC outcomes, the high dropout rate among sirolimus patients makes the practicality of this alternative less convincing. Furthermore, Akker, et al. noted that while sirolimus did prove to have beneficial outcomes at one year, this benefit went away after two years of treatment [26]. Salgo, et al. and Gu, et al. only examining outcomes for one year raise concern regarding the long-term efficacy of sirolimus [24, 27]. Larger scale studies that examine efficacy, adverse events, and dropout rates are indicated to investigate these concerns further.

Campbell, et al. also performed a prospective randomized controlled trial [26]. They examined if conversion from CNI to sirolimus resulted in lower rates of NMSC. Patients were randomized using a computer generated Clinical Operation Randomization Environment schedule. Baseline characteristics were similar between groups. Protocols between the sirolimus and CNI group differed. Patients on CNIs had doses adjusted by study investigators as the investigators felt were best. However, the conversion group had specified loading doses and daily doses, and doses were adjusted as needed to keep drug concentrations within the predetermined ranges. All other aspects of treatment between groups were comparable. Lesions suspicious for NMSC were evaluated and biopsied or excised by dermatologists who were not blinded to treatment group.

The study examined 86 patients, with 39 in the sirolimus group and 47 in the CNI group. Thirty-one of 39 patients in the sirolimus group discontinued treatment, and 23 out of 47 patients in the CNI group withdrew (p = 0.004). Sirolimus group discontinuation was due to the following: 18 from adverse events, 20 from early study termination, one from investigator request, and two from patient request. CNI group discontinuation was due to the following: 19 from early study termination, one from investigator request, one was lost to follow up, and two from patient request. Of note, the difference in withdrawal overall (p = 0.004) and due to adverse events (p < 0.001) between the sirolimus and CNI groups was statistically significant. The attrition rate was thus 79% in the sirolimus group and 49% in the control group. Patients on sirolimus therapy had a significantly shorter length to follow up (0.95 years) than patients on CNIs (1.62 years) due to high rates of study discontinuation in the sirolimus group. The high attrition rate, particularly among the sirolimus group, and the high rate of adverse events raise questions about the practicality and safety of sirolimus conversion. Further, patients who stopped their designated treatment but were still taking part in the study continued to be evaluated for two years.

Intent to treat analysis demonstrated significantly decreased NMSCs per patient per year in the sirolimus group compared to the CNI group (1.31 vs 2.48, p = 0.022). The rates of SCC (0.88 vs 1.77, p = 0.038) were also significantly lower in the sirolimus compared to the CNI group. However, the differences in group protocols, the nonblinded study design, and the high attrition rate should all be considered in the interpretation of these results. No significant difference was found in the BCC groups (0.43 vs 0.77, p = 0.104).

Another randomized trial by Euvrard, et al. looked at sirolimus compared to CNIs in terms of anti-tumoral effect in kidney transplant recipients with previous SCC [27]. To be included in the study, patients had to be kidney transplant recipients with stable renal function on CNIs. They also had to have had at least one post-transplant invasive cutaneous SCC. Patients were randomized in a 1:1 ratio to either continue their current CNI regimen or switch to sirolimus. The article does not specify how the randomization was performed. Sixty-four patients were assigned to the sirolimus group and 56 were assigned to continue CNI therapy. The two groups in the trial were similar in regard to demographics. Fifty-five percent of each group had a history of a single cutaneous SCC at the start of the trial, and 45% of each group had multiple lesions. Both groups were also well-balanced in terms of skin cancer risk factors such as skin type, eye color, age, and sun-exposure. Both groups in this study were treated equally. All patients were examined by a nephrologist and dermatologist at the beginning of the trial and every three months afterward until they had been monitored for two years. During dermatology visits, all skin tumors were recorded and patients were advised about sun protection. During nephrology visits, renal function was followed and immunosuppressive drug doses were recorded. It is unclear whether or not the patients and/or investigators were blinded for this trial. It was also not mentioned whether or not the nephrologists or dermatologists were blind to the study groups. Without this information, it is unclear of the role certain biases may have had in this study.

The results of the study showed that patients in the sirolimus group had a statistically significantly longer survival free of cutaneous SCC than patients in the CNI group. This had a hazard ratio for new SCC of 0.37 (95% confidence interval [CI]: 0.16–0.85), and a study-adjusted hazard ratio of 0.38 (95% CI: 0.17–0.84). In addition to time, patients in the sirolimus group had a decreased percentage of patients who went on to develop a subsequent SCC. Twenty-two percent of patients in the sirolimus group developed a new SCC, and 39% of patients in the CNI group (relative risk, 0.56; 95% CI: 0.32–0.98) developed a new carcinoma. When looking individually at patients with a single carcinoma versus those with multiple carcinomas, the results remain significant only for those with a history of one carcinoma.

While sirolimus led to a greater anti-tumor benefit than calcineurin-inhibitors in terms of cutaneous SCC, it led to more adverse events in patients. Almost every patient that was treated with sirolimus exhibited some form of adverse event related to the drug. Some reactions caused patients to discontinue sirolimus. These events included edema, aphthous ulcers, pneumonitis, exercise dyspnea, and rash. A total of 15 sirolimus patients dropped out from the study due to these adverse effects. The CNI group had three patients drop out due to a diagnosis of cancer. At the end of the two years, 66% of sirolimus patients were still enrolled in the study while 79% of the CNI patients were still enrolled. It is not mentioned in the trial whether or not this difference was significant.

An additional randomized controlled trial was carried out by Akker, et al. in 2013 [28]. They examined whether or not conversion to sirolimus in renal transplant recipients impacted the incidence of SCCs. The study included 155 patients from 21 transplantation centers in the United Kingdom and Netherlands. Patients were randomly assigned to either an experimental (n = 74) or control group (n = 81) and stratified by center, age, and the number of prior SCCs. Patients in the experimental group were converted from azathioprine, mycophenolate mofetil, cyclosporine, and/or tacrolimus to sirolimus. Medication was not altered in the control group. Aside from this intervention, experimental and control group participants were treated equally. Of note, randomization produced comparable groups regarding all patient demographics and baseline characteristics except for sex, but the authors note that primary analysis showed that this discrepancy did not impact results.

Dermatologists examined patients every three months to assess and biopsy lesions suspicious for invasive SCC and BCC. Lesions suspected to be actinic keratosis or SCC in situ were not biopsied. Suspicious lesions were examined by dermatopathologists, and only lesions histologically confirmed to be invasive SCC, in situ SCC, or BCC were included. Dermatologists were blinded to the treatment group; however, the study does not clarify if dermatopathologists were blinded. Patients were not blinded.

The attrition rate of patients regarding completed two-year follow-up was 1.9%. Only three patients did not complete follow-up after two years, and this was due to death during the study period. Thus, intention to treat (ITT) analysis included 72 of 74 patients in the experimental group and 80 of 81 patients in the control group. However, the dropout rate was 52% for the experimental group (n = 39) and 17% for the control group (n = 14), and the authors subsequently performed a per-protocol population (PPP) analysis which examined participants prior to dropout. ITT and PPP analyses were adjusted for age and number of invasive SCCs prior to study participation. Reasons for drop out included death, consent withdrawal, and adverse effects.

Outcomes were assessed dichotomously as the development of SCC among patients one and two years after the study period. Patients converted to sirolimus had a statistically significant lower rate of invasive SCC after one year of treatment [Hazard Ratio = 0.50 (95% CI: 0.28 to 0.90; p = 0.007)]; however, there was a nonsignificant decrease in risk of developing an invasive SCC after two years of sirolimus treatment [Hazard Ratio = 0.76 (95% CI: 0.48 to 1.2; p = 0.255)]. In situ SCC development produced similar findings. The relative risk for SCC in the experimental group was 0.51 (95% CI: 0.32 to 0.82) when stratification by the number of SCCs and age were considered. Conversion to sirolimus also exhibited a nonsignificant decrease in development of BCC [Hazard Ratio = 0.56 (95% CI: 0.30 to 1.1, p = 0.076)].

Overall, sirolimus conversion had no significant impact on SCC development. The inclusion criteria and exclusion criteria (listed in Table 1) suggest generalizability to relatively healthy adults with successful transplantation. Of note, 39% of patients converted to sirolimus experienced adverse events, compared to 2.5% of patients without conversion therapy. The questionable efficacy, as well as the increased incidence of adverse effects, suggests sirolimus conversion may not be ideal. More research is indicated to examine if the adverse effects of sirolimus outweigh the therapeutic benefits.

**Table 1:** Inclusion and exclusion criteria for patients considered for the study. SCC: Squamous cell carcinoma; WBC: White blood cell.

Inclusion Criteria	Exclusion Criteria
1^st^ or 2^nd^ kidney transplant with ≥ one biopsy-confirmed cutaneous invasive SCC Age ≥ 18 years >12 months post-transplantation Stable graft function (estimated glomerular filtration rate ≥20 mL/min weeks before random assignment No acute rejection episode within 12 weeks before random assignment Receiving maintenance calcineurin inhibitor, azathioprine, mycophenolate, and/or steroids for at least 12 weeks before random assignment	Metastatic cutaneous SCC Internal malignancies documented after transplantation Serum creatinine at screening increased >30% above baseline Total WBC count < 3,000/µL Platelet count < 75,000/µL Fasting-triglycerides > 3.95 mmol/L Cholesterol > 7.8 mmol/L (±statins) Transaminases > 2x above normal Planned/present pregnancy Evidence of systemic infection or HIV infection at random assignment

Meta-analyses

Gu, et al. performed a meta-analysis of randomized controlled trials that examined if using sirolimus in post-renal transplantation patients is associated with lower rates of NMSC [29]. Selection criteria proved to be relatively comprehensive. Multiple data bases, annual meetings, relevant journals, and reference lists from relevant papers were reviewed. The search was not restricted by language and unpublished data was searched for by contacting researchers in the field. The authors' search revealed 180 relevant studies from which seven were initially selected, but two were not included. Overall, five studies containing a collective 1499 patients were used in the final analysis. The meta-analysis primarily looked at one-year follow-up; however, if this was not available, data closest to one-year follow-up was used for analysis. The quality of studies was assessed using the Cochrane Risk Bias Tool and the Grading of Recommendations Assessment, Development, and Evaluation principles to assess the strength of individual studies.

Due to heterogeneity between studies, the authors utilized a random-effects model (I2 = 59.8%). Subgroup analysis of BCC demonstrated no heterogeneity (I2 = 0.0%). The authors, however, stated that SCC subgroup analysis demonstrated heterogeneity but reported an I2 = 0.0%. The authors failed to discuss potential causes for heterogeneity. Results were displayed using forest plot, demonstrating that sirolimus treatment resulted in lower rates of NMSC (RR = 0.49, 95% CI: 0.32–0.76, p = 0.001), and this applied to subgroup analysis of BCC (RR = 0.58, 95% CI: 0.37–0.85, p = 0.006) and SCC (RR = 0.58, 95% CI: 0.43–0.78, p < 0.001). Funnel plot analysis indicated no publication bias.

A meta-analysis performed by Knoll, et al. in 2014 sought to examine the effect of sirolimus on the risk of malignancy and death in patients who had undergone renal transplantation [30]. A review of all randomized trials investigating this question was performed. While the primary outcome was the development of any type of malignancy after randomization, there was a secondary outcome which specified the incidence of NMSC. To be included, studies had to compare sirolimus versus other immunosuppressive therapies in the management of renal transplant patients. Trials in which sirolimus was started as the initial post-transplant immunosuppressive agent were examined separately from trials in which another type of immunosuppressant was converted to sirolimus at some point post-transplantation. The authors required the trial to have follow up for at least three months after conversion to sirolimus. It is unlikely that important and relevant studies were missed during the article screening process because the authors searched Medline, Embase and Cochrane from inception to March 19, 2013. Trained reviewers were recruited to screen for articles that matched inclusion criteria. Corresponding authors of eligible trials were contacted and individual patient data was gathered. This included data on malignancy and/or survival. At the end of the screening period, 5876 patients from 21 trials were included in the analysis.

After the analysis was completed, it was determined that 127 (3.8%) patients in the sirolimus group and 116 (4.5%) patients in the control group developed cancer. Overall, when looking at NMSC, there was a 56% reduction in risk for patients taking sirolimus. When only examining NMSC, there was a statistically significant increase in incidence in the control group compared to the sirolimus group (p < 0.001). This remained significant with multivariable adjustment. Of note, this analysis determined that patients in the sirolimus group had a 43% decrease in survival, even after multivariable adjustment (p < 0.001). Of these deaths, there was very few related to malignancy, and of the deaths in each group, malignancy made up a similar proportion.

This review also looked at subgroups within the trials. One subgroup compared conversion trials versus trials in which the patient was started on sirolimus initial immunotherapy. In trials where patients were converted to sirolimus, there was a 68% decrease in the risk of NMSCs (p < 0.001). This contrasted the results of the studies with initial sirolimus therapy which showed no reduced cancer risk in the sirolimus group compared to control (p = 0.65). Another subgroup compared low-dose versus high-dose sirolimus. Both showed a significantly reduced risk of NMSC in patients taking sirolimus versus control (p = 0.006 and p < 0.001, respectively).

Another meta-analysis performed by Yanik, et al. in 2015 also looked at the impact of sirolimus on cancer risk in renal transplant recipients [31]. Compared to the previous meta-analysis, this study performed a literature review using PubMed and also included observational studies in addition to randomized controlled trials. Terms used for the search were “sirolimus AND kidney transplants” as well as “rapamycin AND kidney transplants.” In order to be included, studies had to compare sirolimus to another immunosuppressant in adult kidney transplant recipients. To ensure maximization of the number of patients included, in studies without cancer incidence reported, corresponding authors were contacted for information on cancer incidence that occurred in the trial. If this could not be obtained, the study was not included. To limit the oversight of meaningful, relevant articles, a secondary search of the references and Web of Science was performed which yielded an additional seven articles. After the search was performed and studies that did not meet the criteria were excluded, the final analysis included 22 studies with 39,039 total patients. Of these, 9187 were patients using sirolimus.

The results were then pooled and analyzed. In renal transplant recipients being treated with sirolimus, there was a 29% reduced incidence of cancer when compared to patients being treated with another immunotherapy. NMSC made up a majority of the overall cancer incidence in both groups. When looking at RCTs alone, sirolimus use was associated with a 51% decrease in NMSC incidence (Incidence Rates Ratio = 0.49, 95% CI: 0.32–0.76). For the observational study, a specific association with NMSC was not reported. When combining the RCTs and observation study, there was no overall association between sirolimus use and NMSC risk. It was noted that there were two incidences in which sirolimus therapy was associated with an even stronger reduction in NMSC incidence. One instance was studies that maintained patients on a higher maximum sirolimus target trough level. The other instance was studies that occurred in more recent calendar years.

## Conclusions

There are multiple studies examining if conversion to sirolimus in post-renal transplantation patients can reduce the risk of skin cancer, especially NMSC. A review of the recent literature does not always show consistent conclusions. In some clinical trials or meta-analyses, conversion to sirolimus from CNIs shows promising hope of reducing NMSC and improving survival. However, in other studies, there seems to be no convincing evidence that conversion to sirolimus can significantly decrease the development of NMSC. There may be many factors that contribute to these discrepancies. First, the size of the clinical trial or meta-analysis appears to have an impact. From the selected articles in our review, larger trials and meta-analysis came to the conclusion that conversion to sirolimus in post-renal transplantation patients reduces skin cancer rates, while smaller scale studies tend to yield no clinically significant data. Second, the number of follow-up years may also influence outcomes. Generally, conversion to sirolimus tends to be more effective in reducing NMSC over a short rather than long timeframe. Although there are some inconsistencies in the conclusions, we tend to conclude that conversion to sirolimus reduces NMSC risk in post-renal transplantation patients. Further, there are exciting reports that conversion to sirolimus can even potentially lead to clinical remission in Kaposi’s sarcoma.

One other concern regarding sirolimus conversion is the increased incidence of adverse effects, which can lead to early cessation of sirolimus treatment. It is thus important to determine if the benefits of skin cancer reduction outweigh the adverse effects of sirolimus, and further research may be needed to identify better strategies for reducing these adverse effects and improving overall patient survival. We believe that larger scale clinical trials with better stratification and lower patient dropout rates will allow researchers to draw more definitive conclusions.
